# The impact of beat-to-beat variability in optimising the acute hemodynamic response in cardiac resynchronisation therapy

**DOI:** 10.1016/j.ctrsc.2015.10.004

**Published:** 2015-12

**Authors:** Steven Niederer, Cameron Walker, Andrew Crozier, Eoin R. Hyde, Bojan Blazevic, Jonathan M. Behar, Simon Claridge, Manav Sohal, Anoop Shetty, Tom Jackson, Christopher Rinaldi

**Affiliations:** a Division of Imaging Sciences and Biomedical Engineering, King's College London, UK; b Department of Engineering Science, University of Auckland, New Zealand; c Cardiovascular Department, Guy's and St. Thomas' NHS Foundation Trust, London, UK

**Keywords:** Cardiac resynchronisation therapy, Optimisation, Acute haemodynamic response, Beat to beat variability

## Abstract

**Background:**

Acute indicators of response to cardiac resynchronisation therapy (CRT) are critical for developing lead optimisation algorithms and evaluating novel multi-polar, multi-lead and endocardial pacing protocols. Accounting for beat-to-beat variability in measures of acute haemodynamic response (AHR) may help clinicians understand the link between acute measurements of cardiac function and long term clinical outcome.

**Methods and results:**

A retrospective study of invasive pressure tracings from 38 patients receiving an acute pacing and electrophysiological study was performed. 602 pacing protocols for left ventricle (LV) (n = 38), atria–ventricle (AV) (n = 9), ventricle–ventricle (VV) (n = 12) and endocardial (ENDO) (n = 8) optimisation were performed. AHR was measured as the maximal rate of LV pressure development (dP/dt_Mx_) for each beat. The range of the 95% confidence interval (CI) of mean AHR was ~ 7% across all optimisation protocols compared with the reported CRT response cut off value of 10%. A single clear optimal protocol was identifiable in 61%, 22%, 25% and 50% for LV, AV, VV and ENDO optimisation cases, respectively. A level of service (LOS) optimisation that aimed to maximise the expected AHR 5th percentile, minimising variability and maximising AHR, led to distinct optimal protocols from conventional mean AHR optimisation in 34%, 78%, 67% and 12.5% of LV, AV, VV and ENDO optimisation cases, respectively.

**Conclusion:**

The beat-to-beat variation in AHR is significant in the context of CRT cut off values. A LOS optimisation offers a novel index to identify the optimal pacing site that accounts for both the mean and variation of the baseline measurement and pacing protocol.

## 1 Introduction

Despite continuing efforts, the response rate for cardiac resynchronisation therapy (CRT) remains 50–70% [1], [2], [3]. Ongoing work is focused on optimising lead location [4], [5], [6], endocardial pacing [7], [8], multi-polar pacing [9], [10], [11] and multi-lead pacing [12] to improve response rate. These novel methods of CRT delivery carry promise [13] and it has been suggested that acute measures of contractile response to pacing may help to predict the downstream clinical response. The change in the maximal rate of pressure development (dP/dt_Mx_) within the left ventricle (LV) is a frequently used index of contractility to indicate acute response in both clinical [5], [7], [9], [10], [13], [14], [15], [16] and animal [17], [18], [19] work. However, the utility of this index in identifying long term responders remains controversial [16], [18], [20] and recent results suggest the need for more rigorous methodology and improved transparency in the recording and analysis of this value [21], [22], [23].

The conventional method for reporting the acute hemodynamic response (AHR) to a CRT protocol is the mean percentage change in dP/dt_Mx_, with respect to a baseline measure, in multiple patients with a standard deviation (SD) indicating inter-patient variability. Although providing a broad overview of the changes in response for a given protocol, this approach fails to recognise the beat-to-beat variability within a single protocol. In many cases the use of this approach can be attributed to the nature of the recording software that reports the mean maximal dP/dt_Mx_ over a recorded series of beats and does not readily provide the SD in this measurement.

It is important to note there are numerous physiological and technical reasons why beat-to-beat variability in cardiac contractility exists in the case of these patients. Firstly, ectopic beats are common in heart failure patients [24] and occur with some frequency due to the manipulation of leads and catheters inside the heart. These extra systoles represent an early depolarisation originating out of timing with the intrinsic cycle length. As a result the time for ventricular filling during diastole is altered (compared with the intrinsic sinus rate) and due to Frank–Starling principles, the subsequent cardiac output following an ectopic beat may be increased or reduced. Secondly, respiration alters the beat-to-beat loading on the heart [25] altering filling and local deformation, which both alter cardiac contractility through the Frank–Starling mechanism. Thirdly, patients with atrial fibrillation (AF) have ineffective atrial contractions which can compromise cardiac filling and again alter the contraction on a beat-to-beat basis [26]. Finally, intermittent capture or block will contribute to AHR variation. Note, however, that AHR studies' patients are routinely paced at a fixed rate, removing heart rate variability as a factor.

To investigate the role of beat-to-beat variation in interpreting the AHR, we have developed a software platform (pTool) for offline analysis of pressure transients. This tool enables us to remove spurious ectopic beats from recordings and calculate both the mean and SD of pressure transient phenotypes including dP/dt_Max_, maximal rate of pressure decrease and peak pressure. Here we use this tool to test three hypotheses: Firstly we test if there is significant variation in AHR during CRT pacing protocols. Secondly, we evaluate the confidence that there is a single unique optimal protocol identified by the mean AHR. Thirdly, we test if minimising variation and maximising the mean AHR identifies distinct optimal pacing protocols from solely optimising for mean AHR alone.

## 2 Methods

The study used retrospective data from studies approved by the local ethics committee and informed consent was obtained from each patient. The study population consisted of 38 patients undergoing LV pressure measurements as part of research cases at time of implant and with or without a chronically implanted CRT system. The clinical characteristics of patients studied are shown in Table 1. All patients were treated for dyssynchronous heart failure with CTR. Patients with a mechanical aortic valve or significant peripheral vascular disease were excluded. Baseline assessment included NYHA functional class, ECG and 2D echocardiography prior to the original CRT implant. Each patient's heart failure etiology was confirmed on the basis of clinical history, coronary angiography and/or cardiac magnetic resonance imaging.

### 2.1 Invasive hemodynamic study

The protocol used has previously been described [27]. Patients were lightly sedated using diazepam (5–10 mg). A 0.014 in diameter high fidelity Certus Pressure Wire and PhysioMon software (RADI Medical Systems, Uppsala, Sweden) with a 500 Hz frequency response and 50 Hz filter bandwidth were used to record LV pressure (dP/dt_Max_) [27]; this was passed retrogradely into the LV cavity.

### 2.2 Acute hemodynamic measurement

Pressure recordings were made for 10–20 s for each protocol. The pressure transient recorded during atrial pacing (AAI) or right ventricle (RV) pacing (if the patient was in AF) at 5–10 beats above intrinsic rate was used as baseline. At least 10–15 s was respected after a change in pacing protocol, prior to recording the AHR.

### 2.3 Pressure trace curation

Pressure traces were curated within pTool, a bespoke software platform. Patient data was copied directly from the RADI wire PhysioMon software directory in an ASCII format and read into pTool. Each beat was identified by the minimum pressure value and ectopic beats were selected to be removed from analysis. To remove spurious variations the beat prior to the ectopic beat and for two beats after the ectopic beat were removed as these beats were found to be consistent outliers and lead to over estimation of the AHR variability.

### 2.4 Pacing protocols

Patient data from multiple studies has been combined to maximise the number of pacing protocols evaluated. Patient pacing was performed at 5–10 bpm above intrinsic rate, paced and sensed AV delay of 100 ms and with AAI pacing as baseline. DDD-RV, DDD-LV, DDD-BiV, LV only, LV endocardial, single multipolar LV lead, multiple LV epicardial leads and simultaneous LV epicardial and endocardial lead pacing protocols are included in this study. Capture was verified for each pacing modality by looking for a change in QRS morphology at a paper speed of 200 mm/s.

### 2.5 Statistical analysis

**Fig. 1:**
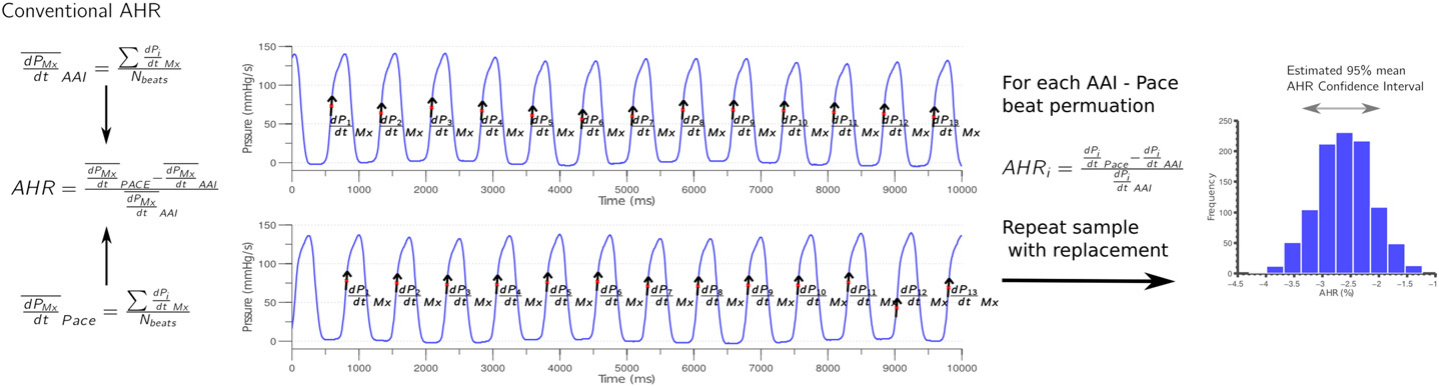
Diagrammatic comparison of conventional mean AHR and bootstrap sampling AHR to estimate mean AHR 95% confidence interval and AHR 95% confidence interval. dP/dt_Mx_ is calculated for each beat for the base line AAI and pacing protocols, generating a set of two dP_i_/dt_Mx_ values (base/paced). Mean AHR takes the mean of the dP_i_/dt_Mx_ values for AAI and pacing protocols and calculates the percentage change. Bootstrapping repeatedly samples pairs from the two dP_i_/dt_Mx_ sets and calculate a set of AHR values the mean and 95% confidence interval are calculated for this set and the process repeated until a family of mean and 95% confidence intervals AHR are generated and the 95% confidence interval of AHR and that of the mean can be determined and plotted.

Fig. 1 provides a schematic for describing the calculation of AHR and beat to beat variability.

To estimate beat-to-beat variation in AHR we consider the empirical distribution of all possible ratios of dP/dt_Mx_ from our protocol and baseline samples and calculate the corresponding AHR for each dP/dt_Mx_ baseline/protocol pair. For each protocol we generate 1000 bootstrap samples by sampling with replacement from this AHR distribution. We calculate the mean and the 5th percentile as the bootstrap replicates. The mean serves as an estimate for the average ratio that would be attained via the protocol, whereas the 5th percentile is a commonly used “level of service” (LOS) threshold — the value which is exceeded by 95% of all ratios attained via the protocol. Using the 5th percentile or mean AHR value *T* from the actual data, and the corresponding 2.5th (*P*_*2.5*_) and 97.5th (*P*_*97.5*_) percentile of the bootstrap replicates, we can calculate a 95% confidence interval for the mean AHR and 5th percentile as [2*T*-*P*_*97.5*_, 2*T*-*P*_*2.5*_]. This allows us to select the optimal protocol for a patient using mean AHR or LOS optimisation. To evaluate the chances of a given protocol being optimal we record, for each of the 1000 bootstrap replicates across all of the protocols, the rank of each of the protocols. The protocol with rank 1 the most times across the 1000 bootstrap samples is deemed best. A clear optimal site was deemed to exist if the difference in times that the best and second best protocol are ranked 1 is greater than 75% of the 1000 bootstraps.

## 3 Results

### 3.1 Beat-to-beat variation in dP/dt_Mx_ during pacing protocols

**Fig. 2:**
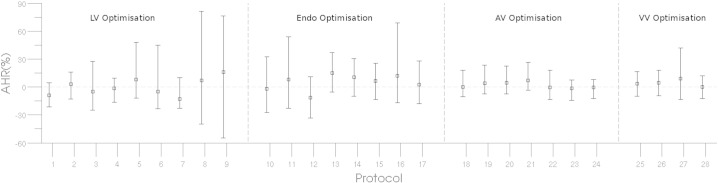
The mean AHR (black box) and the error bars indicate the lower bound and upper bounds of the 95% CI of the 5th and 95th percentile, respectively, for for LV, ENDO, AV and VV protocol optimisation in a single case.

The background beat-to-beat variability in AHR is important when interpreting meaningful changes in contractility with different pacing protocols. Fig. 2 shows the AHR distribution for a single patient receiving LV, ENDO, AV and VV optimisation. In many of the protocol subsets the optimal pacing protocol is clear; however, for protocol 8 and 9 in LV optimisation we see that the variation is sufficiently high that in some beats the response is worse than baseline and these protocols may not represent an effective pacing protocol despite having a high mean AHR.

**Fig. 3:**
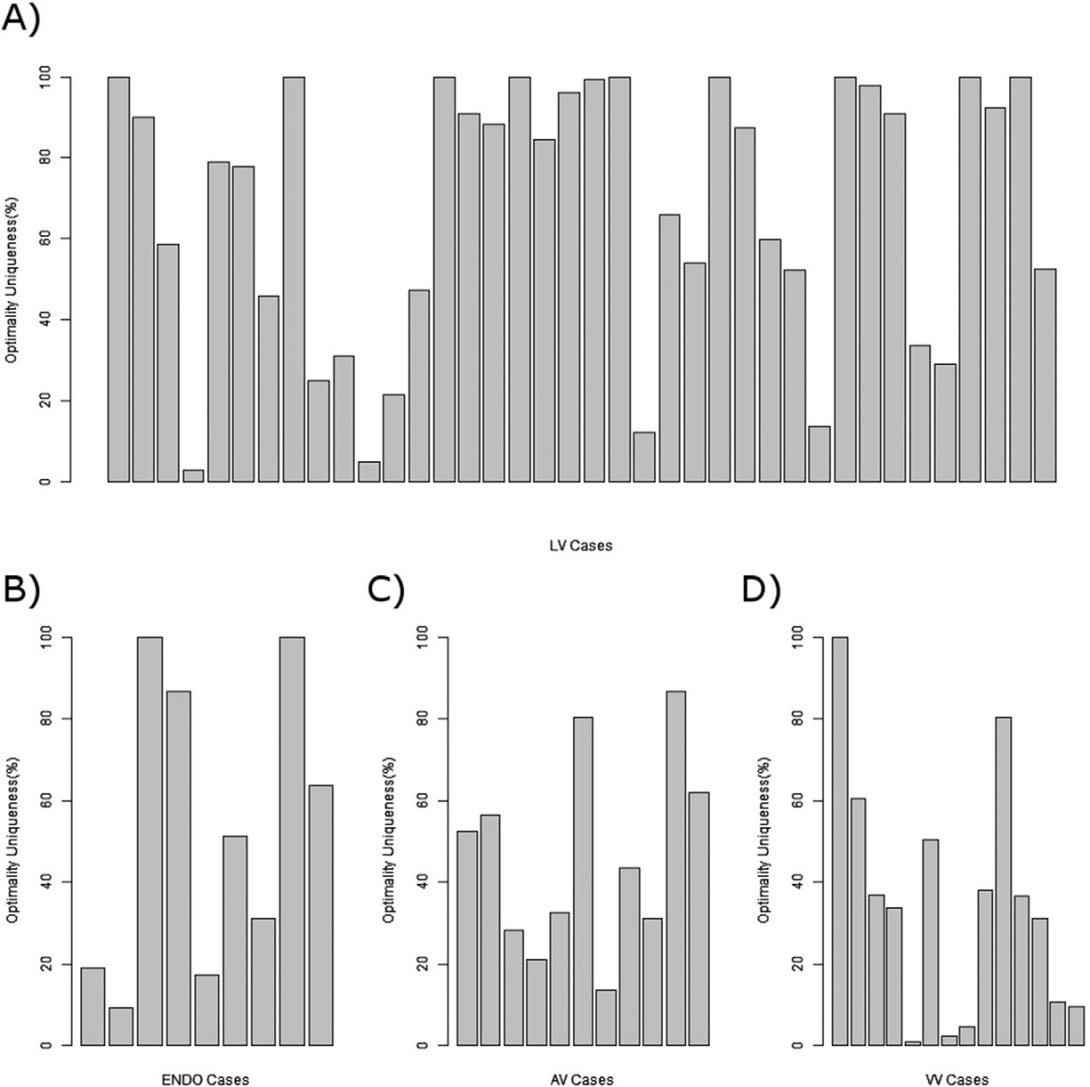
Uniqueness of optimal solution, measured as the difference in likeliness of the two best protocols being optimal, for A) LV, B) ENDO, C) VV and D) AV optimisation cases.

To estimate the variation in response the 95% confidence interval (CI) in AHR for each protocol was evaluated over 1000 bootstrapped samples. Across all 602 protocols evaluated in all 38 patients the mean range of the AHR 95% CI was 6.6%. The mean range of the AHR 95% CI was consistently ~ 7% across LV, AV VV and endocardial optimisation. Considering only the sites that would be selected as optimal based on maximising the mean AHR for each scenario the range of the 95% CI of the mean AHR improvement is 6.4%, 7.6%, 6.4% and 6.2% for LV site optimisation, VV optimisation, AV optimisation and endocardial pacing, respectively (Fig. 3).

### 3.2 Identifying optimal pacing protocols

Optimisation strategies should clearly identify a single optimal pacing site. Comparing mean AHR values provides a single optimal protocol, however, no information is provided on the confidence in or the uniqueness of this solution. Confidence in the optimal site was determined by the number of times a protocol had the highest mean AHR in 1000 bootstrap samples. In 55%, 18%, 14%, and 33% of LV, AV, VV and ENDO optimisation cases, respectively, the confidence in the optimal protocol was greater than 75%. To determine uniqueness of the optimal pacing protocol we compared the confidence in the optimal protocol with the confidence in the next best protocol. Considering all protocols performed on each patient the mean difference between the two top ranking protocols was 57% (min 0.4%). For LV, AV, VV and endocardial optimisation the mean difference was 71% (min 0.4%), 31% (min 5%), 33% (min 4%) and 67% (min 5%), respectively.

### 3.3 Accounting for beat-to-beat variability in measuring response

The specific mechanism through which CRT causes reverse remodelling and improves patient outcome remains controversial. The choice of percentage change in mean AHR as a measure of response does not consider the effect of beat-to-beat variability on patient outcomes. We propose the concept of including the variability in AHR in the measure of acute response using a LOS optimisation.

**Fig. 4:**
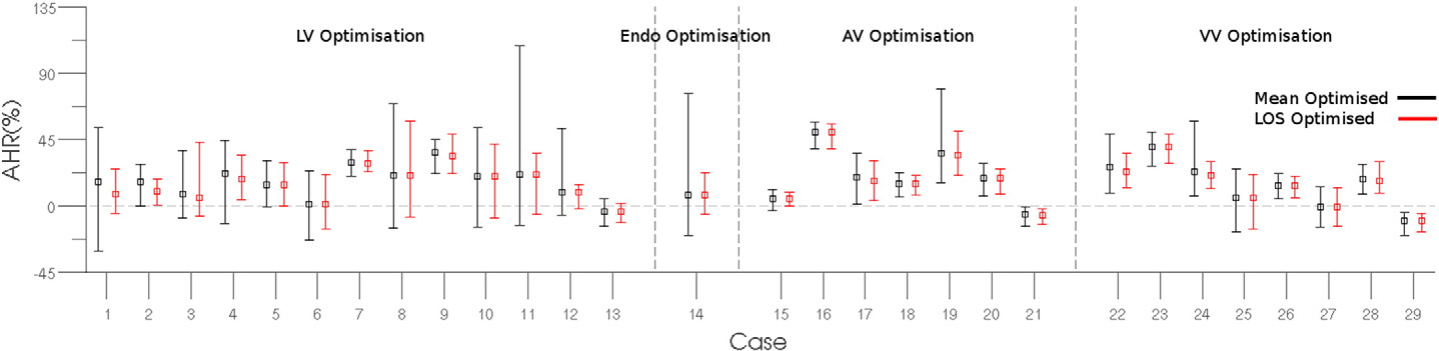
Comparison of mean and confidence interval of the optimal protocol in LV, ENDO, AV and VV optimisation cases selected by level of service (LOS) optimisation (red error bars) or by optimising the mean AHR value (black error bars).

To evaluate if accounting for variability identifies distinct optimal pacing protocols from optimising for the mean AHR response alone we compared which protocol was identified as optimal for the mean AHR and LOS optimisation methods. For LV, AV VV and ENDO optimisation, accounting for variability resulted in different optimal protocols being selected in 34%, 78%, 67% and 12.5% of cases, respectively. Fig. 4 shows the optimal AHR distribution for each case where the optimal site was different between the mean AHR and LOS optimisation.

## 4 Discussion

We have shown that there exists significant beat-to-beat variation in AHR and uncertainty in the mean AHR to pacing. This variation can obfuscate the identification of a single optimal site, device settings or mode of delivery. We found that accounting for variation in measuring the acute haemodynamic response identifies distinct optimal protocols with comparable but more robust AHR.

Despite the use of AHR to characterise the contractile function of the heart, its predictive capacity remains controversial in CRT. We have previously shown that the AHR to pacing is predictive of chronic response with a cut off value of 10% [16], however, in a large retrospective study [18], with different end points, this finding was not borne out. The difference between these findings could be due to the observed variation in AHR, however, this difference could also be compounded by methodological differences between the two studies.

The 95% confidence interval in the mean AHR of 7% is significant in the context of the proposed 10% AHR cut off value for CRT. The uncertainty in the mean value can be reduced through repeated pacing protocol measurements [28] and tightly paired baseline recordings that track the transient movements in patient baseline characteristics [21]. However, these approaches do not account for the inherent beat-to-beat variability that is lost in mean AHR values. High levels of variability may indicate partial capture, intermittent block or may be present due to the physiology of a specific pacing site or activation pattern. Accounting for this variability may be important in improving the predictability of chronic CRT outcomes from acute measurements.

We proposed and evaluated a LOS approach to optimise for the mean and variation of AHR, where a protocol is chosen based on the lower bound of the AHR 5th percentile CI. Optimising to maximise the 5th AHR percentile can be recast as a weighted sum multi objective optimisation problem [29]. For a normal distribution, the 5th percentile is equal to the mean less 1.64 standard deviations. Hence maximising the 5th percentile is approximated by a weighted sum multiple objective optimisation, with the standard deviation having a weighting of − 1.64 times that of the mean value. Here we have used the bootstrap estimate of the LOS index to identify the optimal protocol, which has the benefit of being intuitive, non-parametric and is easy to calculate.

Across LV, AV, VV and ENDO optimisation protocols the mean range of the AHR 95% CI was consistently ~ 7%. Conversely the ability to identify a unique optimal site was lowest for VV and AV optimisation. This may contribute to the lack of clear clinical improvement observed with either AV or VV optimisation [30]. The low uniqueness is potentially explained by the relatively small 10–20 ms differences between protocols. Uniqueness may be improved if a smaller number of protocols with larger differences in delays were evaluated multiple times or for longer periods.

## 5 Conclusion

Variation in the AHR measurement can be substantial during acute CRT pacing studies. Accounting for AHR variation in evaluating optimal protocols may improve the link between AHR and long term CRT outcomes.

## Figures and Tables

**Table 1:** Patient characteristics.

Male	79% (30/38)
Age	67.6 ± 8.2
Ejection fraction	24 ± 7%
Ischemic	53% (20/38)
NYHA	2.6 ± 0.6
QRS duration	143 ± 24
ECG morphology	
LBBB	72% (27/38)
Narrow QRS	19% (7/38)
Non-specific intraventricular conduction delay	6% (2/38)
Right bundle branch block	3% (1/38)
